# Cross-kingdom metabolic manipulation promotes *Salmonella* replication inside macrophages

**DOI:** 10.1038/s41467-021-22198-w

**Published:** 2021-03-25

**Authors:** Deyanira Pérez-Morales, Víctor H. Bustamante

**Affiliations:** Departamento de Microbiología Molecular, Instituto de Biotecnología, Universidad Nacional Autónoma de México, Cuernavaca, Morelos México

**Keywords:** Metabolomics, Infection, Cellular microbiology, Pathogens

## Abstract

Replication inside macrophages is crucial for systemic dissemination of *Salmonella* in hosts. In a *Nature Communications* article, Jiang et al. show that *Salmonella* stimulates glycolysis and represses serine synthesis in macrophages, leading to accumulation of host glycolytic intermediates that the bacteria use as carbon source and as cues for its replication.

## *Salmonella* pathogenicity and SopE2

*Salmonella enterica* serovar Typhimurium (here referred as *Salmonella*) counts with multiple factors and mechanisms to infect humans and other animals^1^. Its pathogenicity was mainly shaped by the acquisition of foreign DNA through multiple horizontal transfer events. In particular, *Salmonella* pathogenicity islands 1 and 2 (SPI-1 and SPI-2) are two gene clusters that were acquired by *Salmonella* at different evolutionary times^2^, each one encoding a Type III Secretion System (T3SS) together with different effector proteins and transcriptional regulators^1^.

A T3SS is a multiprotein complex similar to a syringe, through which bacteria inject effector proteins directly into the cytoplasm of host cells^3^. Effectors injected by the SPI-1 T3SS, including the guanine nucleotide exchange factor (GEF) SopE2, induce cytoskeletal rearrangements and activate signaling cascades that lead to internalization of *Salmonella* into the intestinal epithelium cells, causing enteritis^1^. The resulting intestinal inflammation mediated by SPI-1 effectors, and to some extent also by SPI-2 effectors, generates diverse antimicrobial responses and production of specific compounds that support the growth of *Salmonella*, but not of commensal bacteria, helping the pathogen to outcompete the intestinal microbiota^4^.

The SPI-2 T3SS and its effectors enable the bacteria to replicate inside macrophages^1,5^. In particular, SPI-2 effectors modulate diverse cellular processes to establish the *Salmonella* containing vacuole (SCV), a niche for *Salmonella* replication within macrophages that facilitates evasion of antibacterial responses and efficient acquisition of nutrients^5,6^. Some SPI-1 effectors, including SopE2, also contribute to SCV biogenesis and intracellular replication by unclear mechanisms^7^.

## *Salmonella* induces metabolic alterations in macrophages

**Fig. 1:**
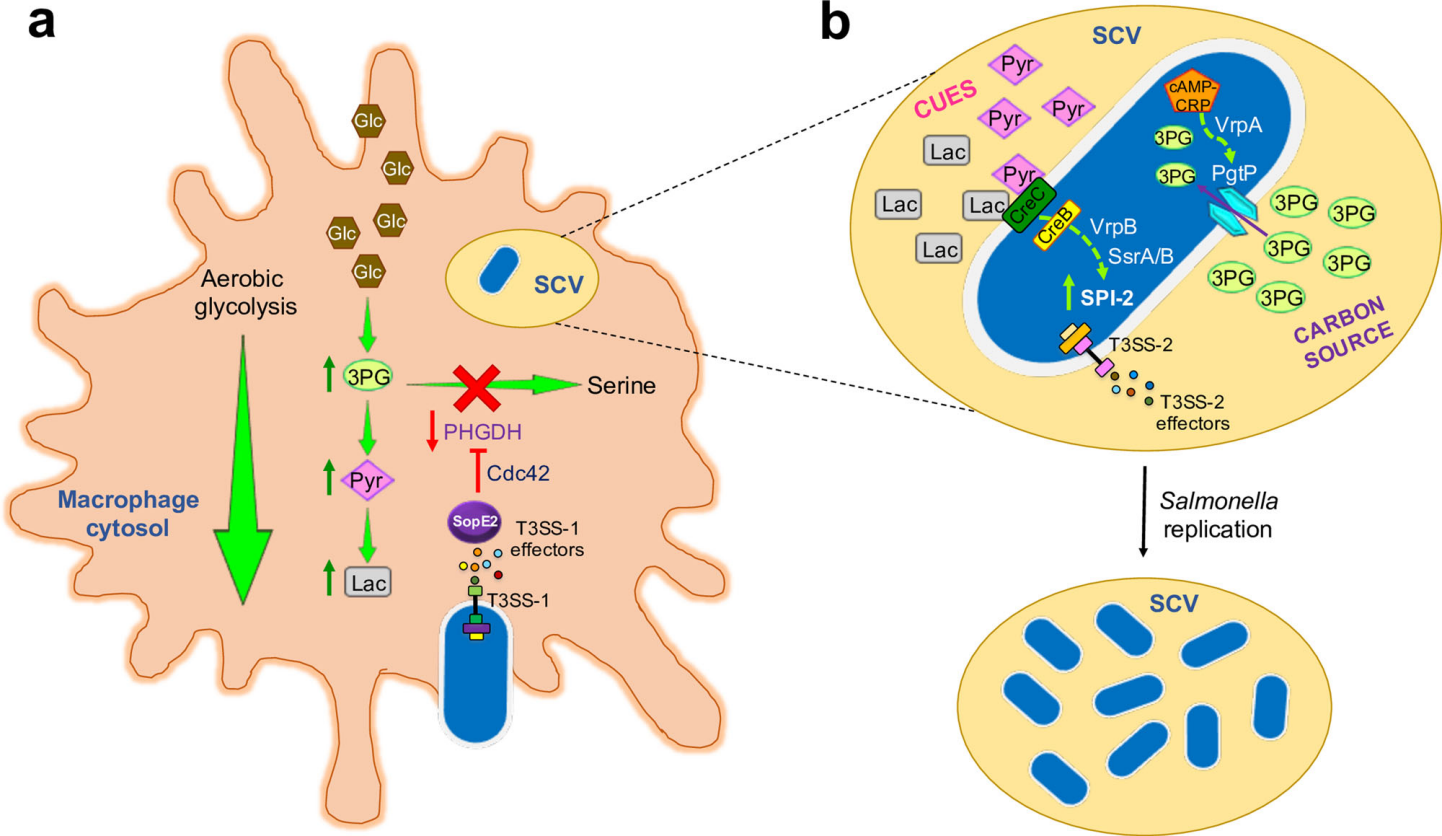
*Salmonella* manipulates macrophage metabolism, thus obtaining a carbon source and cues for intracellular replication. **a** Following infection with *Salmonella*, macrophages increase the conversion of glucose (Glc) to lactate (Lac) through aerobic glycolysis. Additionally, the SopE2 effector, which is injected by *Salmonella* into the cytoplasm of host cells by the SPI-1 T3SS (T3SS-1), represses serine synthesis by inhibiting expression of PHGDH via the host cell Rho GTPase Cdc42. Increased aerobic glycolytic flux and reduction of serine synthesis prompt the accumulation of host 3-phosphoglycerate (3PG), Lac, and pyruvate (Pyr). **b** Within the *Salmonella* containing vacuole (SCV), bacterial replication is supported by the use of host 3PG as a carbon source, while host Lac and Pyr stimulate expression of SPI-2 genes (including those encoding T3SS-2 and effector proteins). In bacteria, the cAMP–CRP complex senses low levels of host Glc and activates the expression of PgtP (3PG transporter) through the VrpA transcriptional regulator. Furthermore, the two-component system CreB/C senses host Lac and Pyr and activates the expression of the VrpB transcriptional regulator, which in turn induces expression of the SsrA/B two-component system, the central positive regulator for SPI-2.

A recent *Nature Communications* paper by Jiang et al^8^. uncovers fascinating new mechanisms mediating *Salmonella* virulence, involving the direct manipulation of macrophage metabolism through effector SopE2 (Fig. 1). Jiang et al. show that *Salmonella* stimulates aerobic glycolysis in macrophages, while the tricarboxylic acid (TCA) cycle and oxidative phosphorylation activities are reduced^8^. This metabolic reprogramming resembles that occurring in proliferating cancer cells, known as the Warburg effect, which transforms most of the incoming glucose to lactate, even under oxygen-rich conditions^9^. It has been proposed that the Warburg effect provides specific nutrients for the multiplication of intracellular bacteria and cancer cells^9^. Indeed, Jiang et al. report that genetic or biochemical inhibition of macrophage glycolysis reduces intracellular replication of *Salmonella*^8^.

Interestingly, the authors also reveal that *Salmonella*-infected macrophages exhibit reduced serine synthesis and diminished activity of downstream metabolic pathways (glycine and glutathione synthesis)^8^. They show that the GEF SopE2 effector and its host target, Rho GTPase Cdc42, inhibit macrophage serine synthesis via downregulation of the *phgdh* gene, encoding a key enzyme in the pathway. Consistently, Jiang et al. report that SopE2 is necessary for effective systemic infection of mice by *Salmonella*, and that both SopE2 and Cdc42 are important for intracellular replication of the bacteria^8^.

The precise mechanisms by which *Salmonella* induces metabolic reprogramming in macrophages, particularly the link between SopE2-Cdc42 and *phgdh* expression, remain yet to be determined. It will also be interesting to understand the potential effects of such metabolic reprogramming on macrophage antimicrobial responses.

## Host metabolites act as nutrients and as signals for the pathogen

Jiang et al. show that the *Salmonella*-induced effects on macrophage metabolism lead to accumulation of host glycolytic intermediates including 3-phosphoglycerate (3PG), a serine synthesis precursor, which *Salmonella* uses as carbon source for replication inside macrophages and during infection of mice^8^.

In addition, the low levels of glucose present in infected macrophages induce upregulation of the bacterial 3PG transporter PgtP, through a regulatory cascade involving the cAMP–CRP complex and a previously uncharacterized transcriptional regulator, VrpA^8^. Thus, the alterations in macrophage metabolism not only provide *Salmonella* with a carbon source (3PG) but also with a cue (low glucose) that triggers the uptake of 3PG for intracellular replication.

The authors show that increased glycolysis within macrophages leads to accumulation of pyruvate and lactate, which also promote intracellular replication of *Salmonella* by stimulating SPI-2 gene expression^8^. The mechanism for this upregulation of SPI-2 is mediated by the two-component system CreB/C (known to be activated by pyruvate, lactate, and other short-chain carbon sources^10^), together with a previously uncharacterized regulator (VrpB) and the SsrA/B two-component system (a known positive regulator of SPI-2^1^)^8^.

Jiang et al. also report succinate accumulation within infected macrophages^8^. Interestingly, a recent study has shown that increased succinate levels in *Salmonella*-infected macrophages induce expression of SPI-2 genes and of genes associated with antimicrobial resistance^11^. We wonder whether succinate might activate SPI-2 expression through the CreB/C-VrpB regulatory cascade described by Jiang et al^8^. Interestingly, it is known that *Salmonella* utilizes microbiota-derived succinate and host-derived lactate as carbon sources to efficiently colonize the gut^12,13^.

All these findings suggest that *Salmonella*, and probably other bacteria, reprogram their own metabolism and regulatory mechanisms to take the best advantage of metabolites present in different niches of their hosts, using them as nutrients and/or cues.

## Concluding remarks

The study by Jiang et al^8^. reveals novel mechanisms mediating *Salmonella* pathogenesis and illustrates some remarkable strategies developed by bacteria to adapt and survive in their hosts (Fig. 1). Other intracellular pathogenic bacteria such as *Brucella abortus*, *Chlamydia pneumoniae*, *Chlamydia trachomatis*, *Legionella pneumophila*, and *Mycobacterium tuberculosis* are also known to shift their host cells towards a Warburg-like metabolism (aerobic glycolysis)^9^. This metabolic reprogramming of host cells seems to be pathogen-specific^9^, but the mechanisms behind it are still poorly understood. We expect that future research in this area will provide new strategies to develop anti-infective therapies.
